# Global Health Diplomacy, Monitoring & Evaluation, and the Importance of Quality Assurance & Control: Findings from NIMH Project Accept (HPTN 043): A Phase III Randomized Controlled Trial of Community Mobilization, Mobile Testing, Same-Day Results, and Post-Test Support for HIV in Sub-Saharan Africa and Thailand

**DOI:** 10.1371/journal.pone.0149335

**Published:** 2016-02-22

**Authors:** Sebastian Kevany, Gertrude Khumalo-Sakutukwa, Basant Singh, Alfred Chingono, Stephen Morin, NIMH Project Accept Study Team

**Affiliations:** 1 University of California San Francisco, San Francisco, California, United States of America; 2 Medical University of South Carolina, Charleston, South Carolina, United States of America; 3 University of Zimbabwe, Harare, Zimbabwe; University of Malaya, MALAYSIA

## Abstract

**Background:**

Provision and scale-up of high quality, evidence-based services is essential for successful international HIV prevention interventions in order to generate and maintain intervention uptake, study integrity and participant trust, from both health service delivery and diplomatic perspectives.

**Methods:**

We developed quality assurance (QAC) procedures to evaluate staff fidelity to a cluster-randomized trial of the NIMH Project Accept (HPTN 043) assessing the effectiveness of a community-based voluntary counseling and testing strategy. The intervention was comprised of three components—Mobile Voluntary Counseling and Testing (MVCT), Community Mobilization (CM) and Post-Test Support Services (PTSS). QAC procedures were based on standardized criteria, and were designed to assess both provider skills and adherence to the intervention protocol. Supervisors observed a random sample of 5% to 10% of sessions each month and evaluated staff against multiple criteria on scales of 1–5. A score of 5 indicated 100% adherence, 4 indicated 95% adherence, and 3 indicated 90% adherence. Scores below 3 were considered unsatisfactory, and protocol deviations were discussed with the respective staff.

**Results:**

During the first year of the intervention, the mean scores of MVCT and CM staff across the 5 study sites were 4 (95% adherence) or greater and continued to improve over time. Mean QAC scores for the PTSS component were lower and displayed greater fluctuations. Challenges to PTSS staff were identified as coping with the wide range of activities in the PTSS component and the novelty of the PTSS process. QAC fluctuations for PTSS were also associated with new staff hires or changes in staff responsibilities. Through constant staff monitoring and support, by Year 2, QAC scores for PTSS activities had reached those of MVCT and CM.

**Conclusions:**

The implementation of a large-sale, evidence based HIV intervention requires extensive QAC to ensure implementation effectiveness. Ongoing appraisal of study staff across sites ensures consistent and high quality delivery of all intervention components, in keeping with the goals of the study protocol, while also providing a forum for corrective feedback, additional supervision and retraining of staff. QAC ensures staff fidelity to study procedures and is critical to the successful delivery of multi-site HIV prevention interventions, as well as the delivery of services scaled up in programmatic situations.

## Background

The heightened profile of global health efforts in the international relations and political spheres in turn requires enhanced efforts to ensure that programs are delivered in the most adaptable, appropriate and diplomatic manner manner. In this context, there remains no more severe global health crisis in the world today than the HIV epidemic in sub-Saharan Africa. There are approximately 25 million people living with HIV in sub-Saharan Africa, with 1.6 million new infections occurring in 2012 alone.[1] In addition to the global death and disease burden, the epidemic has had an enormous impact on economies and life expectancies, and left a legacy of millions of orphans. Structural factors, such as economic, social, legal and cultural conditions also contribute to increased risk for HIV infection in sub-Saharan Africa.[1]

In response, with associated reference to concentrated HIV epidemics in countries such as Thailand, the global health community has mobilized unprecedented levels of resources in a concerted attempt to turn the tide of the epidemic.[1] This has elevated the role of global health to the realm of ‘high politics, in which both diplomatic and health outcomes of interventions must be considered,[2] leading to the development of diplomatic perspectives on global health interventions, such as the ability of staff and programs to pursue international relations goals. In this context, the provision of Voluntary HIV Counseling and Testing (VCT) services, as part of the comprehensive approach to HIV prevention, has been established as a key response to the epidemic.[3] Such HIV testing is linked with clinical and community interventions, improved referrals to care, treatment, prevention, and post-test support services.

### Project Accept

Project Accept (HPTN 043) a cluster-randomized trial of community mobilization, mobile testing, same-day results, and post-test support for HIV was conducted in Sub-Saharan Africa and Thailand. The intervention, described in detail elsewhere[4] focused on the ‘scaling-up’ of evidence based HIV treatment and prevention strategies on an unprecedentedly large scale, covering four countries with severe generalized or concentrated HIV epidemics. Briefly, 34 communities in Africa (in South Africa, Tanzania, and Zimbabwe) and 14 communities in Thailand were randomized to receive either a community-based voluntary HIV counseling and testing (CBVCT) intervention in addition to standard clinic-based VCT (SVCT) services, or SVCT services alone.

The CBVCT intervention had three major strategies: (1) To make VCT more available in community settings via mobile voluntary counseling and testing (VCT); (2) to engage the community through outreach via community mobilization (CM); and (3) to provide post-test support services (PTSS), irrespective of the participants’ HIV status. For mobile VCT, mobile units providing HIV testing were available at a range of venues throughout intervention communities for the duration of the three-year intervention period. For CM and PTSS, specific aims were to (1) create awareness about and open dialogue around HIV/AIDS in communities, (2) enhance the communities’ understanding of, participation in, and enthusiasm for MVCT, (3) foster understanding and acceptance of HIV positive members of the community (stigma reduction), and (4) promote HIV risk reduction among all community members. The intervention is designed to be evidence-based, cost-effective, and feasible for rapid scale up in resource-poor settings.[4, 5]

These strategies were designed to change community norms and reduce risk for HIV infection among all community members, irrespective of whether they participated directly in the intervention. In addition, given the broad range of cultural geographical, religious and social context in which these interventions were designed and delivered, these strategies were designed with close attention to diplomatic considerations, including the maintenance of appropriate standards of behavior and service delivery standards by project staff. The intervention lasted for three years in each site.

### A Multilevel Prevention Intervention

Project Accept is characterized as a multicomponent, multilevel prevention intervention. The multilevel prevention framework has roots in the “ecological model,” understanding the individual as embedded in societal, community, familial, and peer contexts and posits that behavior is shaped by economic, political, and social structures; sociocultural contexts; and social relationships in which people negotiate behaviors. As a result, multilevel interventions aim to address the multiple structural or sociocultural factors that influence an individual; these include interpersonal processes, community factors, and institutional factors. In addition, given the broad range of cultural geographical, religious and social context in which these interventions were designed and delivered, these strategies were designed with close attention to diplomatic considerations, which included maintenance of partnerships with stakeholders at all levels within the community, administrative, and political structures, thereby facilitating scale-up across diverse settings and broader geographical areas. The intervention also ensured appropriate standards of behavior and high quality of service delivery by project staff. All participants in the PTSS and MVCT arms of the study provided written consent. Due to the community-based nature of the CM component, community-based acceptance was attained in advance of related activities by relevant social and political leaders.

### Quality Assurance for HIV Prevention Interventions

Provision of high quality, diplomatically-sensitive services is essential for successful HIV prevention interventions. The development of efficient and effective procedures designed to monitor the quality of service delivery is therefore central to study planning and implementation. The treatment integrity of psychotherapy interventions, which describes the degree to which an intervention is delivered as intended, was found to be adequately addressed in only 3.5% of evaluated interventions, though no related work has been undertaken for HIV/AIDS interventions specifically.[6] A number of recommendations have been provided in the literature on the implementation of treatment integrity procedures.[7–11]

Quality Assurance (QAC) is defined as the steps taken in advance to increase the quality and consistency with which an intervention is conducted.[12] Quality Control (QC) consists of activities conducted when the intervention is in the field in order to quickly identify and correct deviations from protocol as well as identify, according to standard operating procedures, sub -optimal performance (e.g. errors in staff judgment; non-adherence to study process; participant problems) Both QAC and QC procedures (hereafter referred to as ‘QAC’) are designed to maintain the integrity of the components by assessing adherence and assisting staff in meeting these goals, and are essential components in ensuring maintenance of quality control for “scaled-up” evidence-based interventions. Perhaps most importantly, the most essential overarching goal of the QAC process, besides maintaining study integrity, is to generate and maintain intervention uptake, and participant trust.[13] In this paper, we describe the methodology, results and effects of QAC monitoring throughout three years of Project Accept activities in intervention communities.

## Methods

### Ethics Statement

This research was approved by the U.S. National Institute of Mental Health as a cooperative agreement, through contracts U01MH066687 (Johns Hopkins University), U01MH066688 (Medical University of South Carolina), U01MH066701 (University of California, Los Angeles), and U01MH066702 (University of California, San Francisco). In addition, this work was supported by the HIV Prevention Trials Network (HPTN Protocol 043) of the Division of AIDS of the U.S. National Institute of Allergy and Infectious Diseases, and by the Office of AIDS Research of the U.S. National Institutes of Health. Institutional review board (IRB) approval was obtained from all US and international sites, including UCSF's Committee on Human Research and all other relevant ethics committees. All participants in the post-test support services (PTSS) and mobile voluntary counseling and testing (MVCT) arms of the study provided written consent. Due to the community-based nature of the community mobilization (CM) component, community-based acceptance was attained in advance of related activities by relevant social and political leaders.

**Fig 1:**
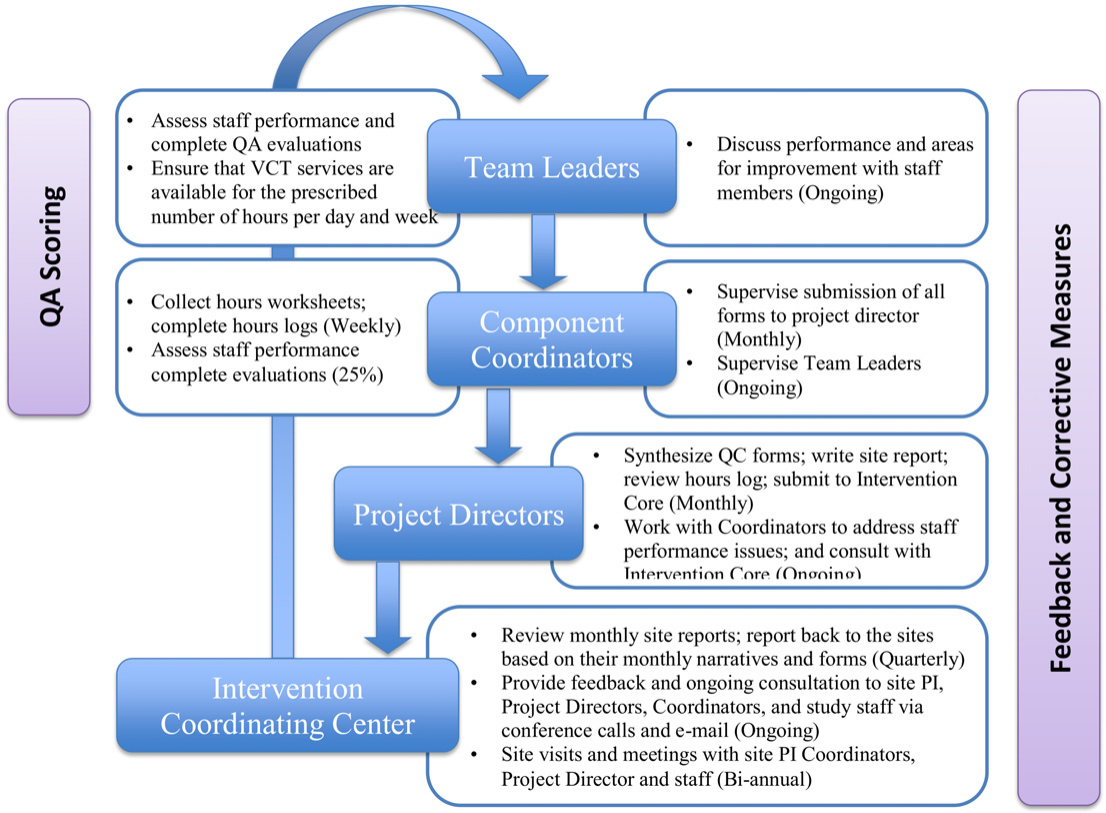
Quality Assurance & Control Reporting System and Responsibilities.

The QAC reporting system and responsibilities are outlined in Fig 1 (Quality Assurance & Control Reporting System and Responsibilities). In order to ensure that QAC criteria were applied consistently across sites, a comprehensive, centralized 8- day Training of Trainers (TOT) meeting was conducted for project managers and component coordinators at the outset of the intervention. The TOT included discussion of the theoretical underpinnings of the intervention, a detailed review of QAC procedures, review of the rating criteria and operational definitions, and practice QAC sessions and discussion. In addition, all relevant study staff were provided with extensive training on the importance of adapting the Project Accept intervention to local conditions, with appropriate awareness of local cultural, religious and social norms. All study staff (counselors, research nurses, outreach workers and team leaders) were subsequently trained by study coordinators at their respective study sites. All study staff were also trained in “Good Clinical Practice” (GCP), provided by the HIV Prevention Trials Network (HPTN).

Study coordinators were required to hold at least a master’s degree in social sciences as well as supervision experience. All other study staff (counselors, research nurses and outreach workers) were required to have a minimum of a diploma in their professional area of expertise. In addition, they received Project Accept training in HIV counseling (study counselors); HIV counseling and testing and Rapid testing (research nurses); HIV counseling and group facilitation (PTSS staff); and community outreach and education techniques (CM outreach workers). Team leaders appointed for each of the study components received additional training in basic management, supervision and support skills. For each study component, training was standardized across sites using training manuals developed by Project Accept [http://hivinsite.org/accept].[14] Efforts were made to ensure the trainings provided matched expected in-country and international standards and guidelines. From a diplomatic perspective, individuals who proved their ability to establish rapport, demonstrate good listening skills, and be supportive, respectful, and nonjudgmental, as well as demonstrating proficiency in basic counseling skills (active listening, reflection, and information gathering), and the basic facts of HIV transmission, were chosen as counselors. New staff were selected and trained by the site project director or a designated senior staff trainer. These procedures helped to ensure that identical procedures and content were delivered across study sites.

QAC roles and responsibilities at the site level are presented in Fig 1. Consistent with recommendations for QAC review of behavioral and psychological interventions (Waltz et al, 1993), ratings were made for both (1) adherence to protocol and (2) competence at conducting the intervention. Standard operating procedures (SOP) manuals detailed QAC procedures for each intervention component and outlined step-by-step implementation procedures including goals, materials required, and timelines. Team leaders and coordinators monitored staff performance in the VCT, PTSS, and CM intervention components via (1) weekly supervision of field staff by team leaders and (2) independent review and rating of VCT, CM and PTSS sessions. Team leaders observed sessions conducted with participants in the field, and rated their fidelity to the protocol on multiple essential components using QAC evaluation forms scored with a 5-point scale. Supervisors observed a random sample of 5% to 10% of sessions each month and evaluated staff against multiple criteria on scales of 1–5. A score of 5 indicated 100% adherence, 4 indicated 95% adherence, and 3 indicated 90% adherence. Scores below 3 were considered unsatisfactory, and protocol deviations were discussed with the respective staff. Component coordinators were asked to evaluate at least 25% of sessions at their discretion to ensure both (1) inter-rater reliability and (2) that QAC activities were both timely and accurate.

### Reliability & Validity of QAC Measures

For project-specific QAC measures to be effectively interpreted and generalized, it is essential that reliability and validity of related scales be considered. In the case of Project Accept, measurement of key constructs (e.g. PTSS protocol adherence) was validated during QAC tool development, based on a collaborative process with both service delivery staff and the input and approval of clients [4]. For example, the use of specific scales (e.g. QAC scores of 1 to 5) was based on approvals and inputs from both QAC supervisors and project staff on the basis of comparability and user-friendliness. Of equal importance in this regard are the links between QAC scales and scores and primary intervention outcomes (e.g. number of HIV/AIDS infections averted). In order for QAC scores to be both valid and reliable in this regard, links between positive health outcomes and high QAC scores (allowing for relevant confounders) is critical. In this regard, though beyond the scope of this paper, comparison with the final outcomes and impact of the Project Accept intervention will allow for the establishment (if any) of such links.

### Sampling Strategy

Throughout the intervention, a random sample of VCT, CM and PTSS sessions were selected for QAC evaluation by team leaders. For VCT, during the first 6 months of the intervention 10% of counseling sessions were evaluated, with a minimum of 2 evaluations per counselor per month. Thereafter, 5% of sessions delivered per month were evaluated with a minimum of 1 evaluation per counselor per month. For CM, during the first six months 15% of outreach sessions were evaluated, with a minimum of 2 evaluations per CM staff member per month. Thereafter, 5% of outreach sessions were evaluated, with a minimum of 1 evaluation per CM staff member per month. For PTSS, during the first 6 months of the intervention all CET and stigma reduction sessions, 2 information sharing sessions, 20% of support sessions and 15% of crisis counseling sessions were evaluated. Thereafter, 1 CET session, 1 stigma reduction session, 4 information sharing sessions, 5% of support sessions and 5% of crisis counseling sessions were evaluated.

### Evaluating Intervention Components

For the VCT component, VCT team leaders assessed counselors’ adherence and skill levels in 10 essential component areas, including (1) general counselor skills in keeping with the client-centered, personalized risk reduction model recommended by the U.S. Centers for Disease Control and Prevention (CDC) and World Health Organization (WHO)[15] and (2) adherence to counseling strategy. Each of these component areas were then assessed via approximately 10 required activity or skill criteria. General counselor skills included empathy, being non-judgmental, maintaining appropriate boundaries, and relaying the objectives of the VCT session. As with CM and PTSS, counseling staff were required to review component or session protocols, session checklists, and any other relevant information in advance of each session. From a diplomatic perspective, QAC procedures (1) helped to ensure that project staff completed the VCT process in a non-threatening and sensitive fashion and (2) in accordance with the expected international standards for the delivery of HIV counseling and testing.

For the CM component, CM team leaders assessed outreach workers’ adherence and skill levels for 10 essential areas of community interaction and 6 areas of community referral and follow-up. These included (1) basic interaction skills, (2) provision of accurate information, and (3) referrals to further care. Interaction skills included empathy, being non-judgmental, maintaining appropriate boundaries, and maintaining session cohesion. In addition to adherence to manualized guidelines, CM activities were also rated, using the same scales, on the skill with which the staff member delivered the session. In particular, community mobilization staff were assessed on their success in adapting related activities to observe local social, cultural and religious norms,[16] thereby ensuring that the Project Accept intervention was delivered to recipient communities and individuals with appropriate attention to diplomatic considerations.

For PTSS, team leaders assessed facilitators’ adherence and skill levels in individual crisis counseling, group information sessions, coping effectiveness training, and stigma reduction training (each with approximately 12 activity or skills criteria). Staff were evaluated on (1) basic support service delivery skills, (2) skills in guiding goal-setting, and (3) adherence to the PTSS curriculum. PTSS activities were evaluated and scored separately. As with CM and VCT assessment procedures, PTSS staff and service delivery was assessed with reference to acceptable standards of service delivery from the diplomatic perspective, including the provision of long-term, sustainable and effective coping strategies for patients diagnosed with HIV.

### QAC Scoring System

For each session, project staff were informed in advance of QAC assessment in advance. While this generated a risk that QAC scores would be higher when staff were aware that the specific sessions were being assessed, given staffing and spatial constraints, alternative blinded or covert assessment systems were considered unfeasible by the Intervention Core. Team leaders then recorded the relevant QAC score for each evaluation criterion on the appropriate QAC form. Evaluation criteria were thematically grouped by essential component area, and the average score for each group was recorded at the end of each section. Individual item scores within an area were averaged to create a summary score and those summary scores were again averaged for an overall QAC score. At the end of the form, a single overall score for the entire session was calculated based on an average of these scores. For each intervention component, these scores were then aggregated and averaged to generate a single QAC score for VCT, CM, and PTSS each month. At the end of each month, all QAC records for each intervention component across sites were transmitted to the intervention coordinating center via scanned, security-protected e-mail documents. These reports were accompanied by a narrative report from the site project director describing QAC activity over the reporting period. Receipt of QAC data was acknowledged, entered into a password-protected Microsoft Access database, and cross-checked through Microsoft Excel.

### Feedback and Corrective Measures

QAC performance charts across intervention components were produced for each study site by the intervention coordinating center and returned to site project directors with commentary and questions on performance on a monthly basis. Feedback reports also described emerging QAC issues across study sites in order to maintain communication and interactive learning between sites. Study sites were encouraged to use these reports as a training tool to focus attention on specific QAC issues, provide early feedback, and foster preventive measures. QAC data was also presented and examined on a monthly basis with US and site-based principal investigators via conference calls with the study steering committee.

Also at the site level, feedback mechanisms included (1) weekly field staff meetings and (2) periodical written feedback to field staff by team leaders, component coordinators and project directors. QAC issues were also discussed at component-specific staff meetings, study-wide staff meetings, and in one-on-one meetings with project staff. The frequency and structure of these meetings was left to the discretion of the site team leaders and coordinators, who were judged to have the best assessment of staff needs. Team leaders and component coordinators used the QAC scores to (1) identify strengths and weaknesses of individual staff and overall areas and components of the intervention and (2) create action plans for improvement, retraining, or more frequent supervision and support. Staff failing to meet minimum QAC requirements were retrained and only allowed to resume their role after conducting 2 observed and evaluated sessions rated at 90% adherence or higher.

In addition, all study sites received support and monitoring visits from the intervention coordinating center at UCSF and designated National Institutes of Mental Health (NIMH) staff. In this context, monitors accessed and inspected study facilities and documentation, as well as observing the performance of study procedures. In addition, the intervention coordination director visited each study site bi-annually to evaluate the quality and consistency of the implementation of the intervention. These visits allowed firsthand observation of the intervention components and maintained open relations with field staff. Study site visits generally took one week to conduct and included: (1) Observing sessions and rating performance using the QC evaluation form; (2) reviewing files and forms for completeness and accuracy; (3) observing daily operations; (4) ensuring that the intervention was being implemented as prescribed in the Protocol and SOP guidelines; and (5) individual and group meetings with the site teams (site PI, project director, coordinators and staff) to provide direct supervision, feedback and answer questions. Study site visits were supported by monthly conference calls with each site.

## Results

### VCT

**Fig 2:**
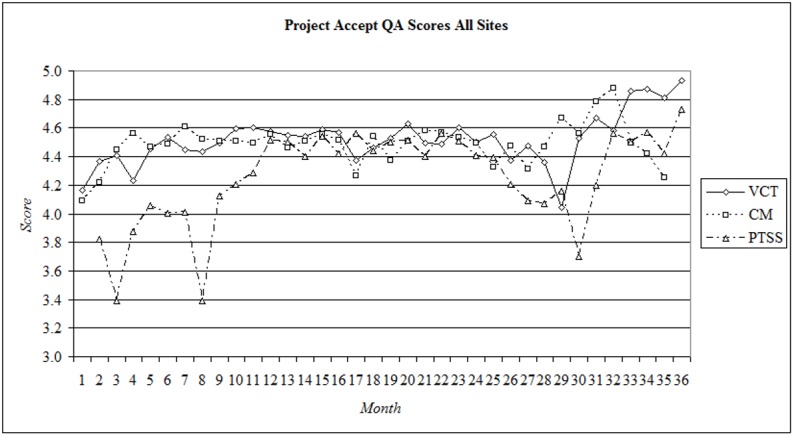
Quality Assurance & Control Scores for All Sites.

**Fig 3:**
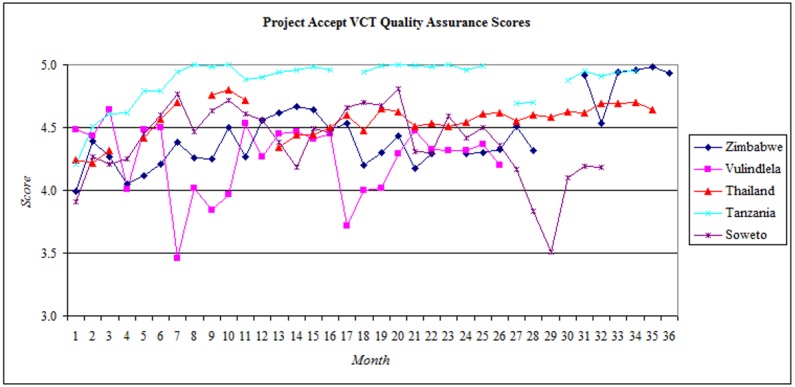
VCT Quality Assurance & Control Scores by Site.

QAC scores across all components are presented in Fig 2 (Quality Assurance & Control Scores for All Sites) and VCT QAC scores are presented in Fig 3 (VCT Quality Assurance & Control Scores by Site). Periodic breaks in intervention delivery for holidays and rest periods are displayed as gaps in the chart data. All sites began the intervention with VCT QAC scores between 3.9 and 4.5. In Soweto, VCT QAC scores rose consistently throughout the first 6 months and remained at high levels throughout Year 1 and Year 2. In Year 3, scores declined sharply to 3.5, but recovered in succeeding months. In Tanzania, VCT QAC scores rose steadily during Year 1 of the intervention, and remained at a high level throughout Year 2. VCT QAC scores fell to 4.7 in Year 3. In Thailand, VCT QAC scores were consistently high during Year 1 and Year 2, although the cyclical nature of intervention service delivery there meant that VCT QAC data was not collected in all months. In Vulindlela, VCT QAC scores fell sharply during Year 1 (3.5) and Year 2 (3.7). In Zimbabwe, VCT QAC scores remained between 4.0 and 4.7 throughout Year 1 and Year 2. In Year 3, after a prolonged absence from field work due to national elections, VCT QAC performance was above 4.5 and remained high throughout the remainder of the intervention. Overall, during Year 1 of the intervention the mean VCT QAC scores across the 5 study sites were 4 (95% adherence) or greater and remained consistently high during Year 2, with some fluctuations. During Year 3, VCT QAC scores fell to 4 and then recovered to levels above 4.5 throughout the final six months of the intervention.

### CM

**Fig 4:**
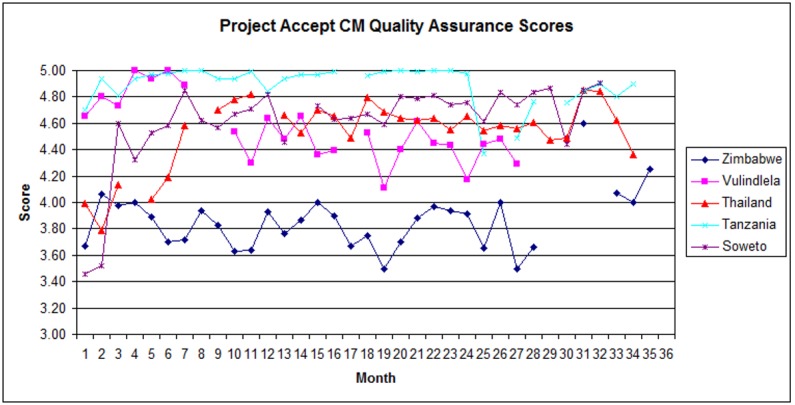
Community Mobilization Quality Assurance & Control Scores by Site.

CM QAC scores are presented in Fig 4 (Community Mobilization Quality Assurance & Control Scores by Site). CM QAC scores began at between 3.4 (Soweto) and 4.7 (Tanzania). In Soweto, CM QAC scores rose quickly during the initial months of the intervention from 3.5 to 4.8, and only declined to 4.4 in Year 3. In Tanzania, CM QAC scores remained consistently high throughout Year 1 and Year 2 before declining to 4.4 in Year 3. CM QAC scores improved throughout the remainder of Year 3, and the site finished the intervention with average scores above 4.8. In Thailand, CM QAC scores rose from 4.0 to 4.8 throughout Year 1, and remained at high levels throughout Year 2 before declining to 4.5 in Year 3. In Vulindlela, CM QAC scores fell periodically during Year 1 (to 4.3) and Year 2 (to 4.1). The site’s CM QAC scores stabilized in Year 3. In Zimbabwe, CM QAC scores were lower than at other sites throughout Year 1 and Year 2, with a range of 3.7 to 4.1, but remained at acceptable levels and, as with VCT QAC, improved during Year 3 (to 4.8) after an enforced absence from fieldwork. Overall, during Year 1 months of the intervention, the mean scores of CM staff across the 5 study sites were 4 (95% adherence) or greater and continued to improve over time. CM scores remained consistently high throughout Year 2 of the intervention and rose again during the first half of Year 3 to 4.8 before declining to 4.2 in the final months of the intervention.

### PTSS

**Fig 5:**
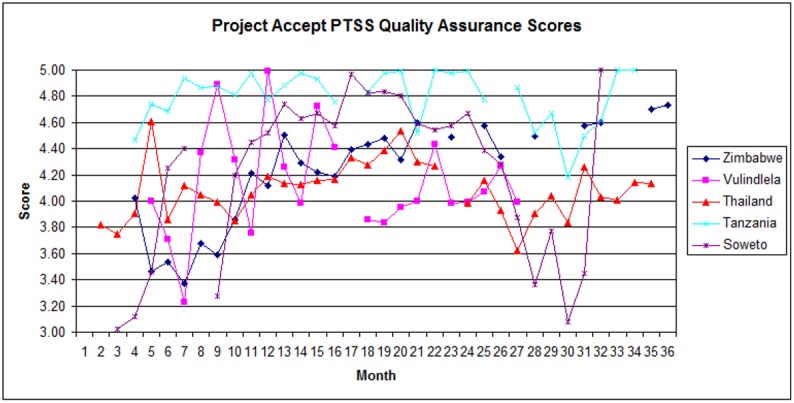
Post-Test Support Services Quality Assurance & Control Scores by Site.

PTSS QAC scores are presented in Fig 5 (Post-Test Support Services Quality Assurance & Control Scores by Site). In keeping with the intervention protocol, PTSS activities were not initiated in sites until between the second and fifth month of the intervention. As compared to VCT and CM, there was a wider range of initial QAC scores across sites, varying from 3.0 in Soweto to 4.5 in Tanzania. In Soweto, PTSS QAC scores rose rapidly during the first half of Year 1, from 3.0 to 4.4, before declining to 3.3. PTSS QAC scores then rose consistently throughout Year 2. In Year 3, PTSS QAC scores fell to 3.0 before recovering. In Tanzania, PTSS scores rose throughout Year 1, from 4.5 to 5.0, before declining briefly to 4.8 during Year 2. In Year 3, PTSS QAC scores fell again to 4.2, but recovered to levels above 4.8 by the end of the intervention. In Thailand, PTSS QAC scores improved rapidly during the first months of Year 1, from 3.8 to 4.6, before declining to 3.9. PTSS QAC scores remained at high levels throughout Year 2 before declining to 3.6 in Year 3. The site finished the intervention with a mean score of 4.0. In Vulindlela, PTSS QAC scores fluctuated throughout Year 1 and Year 2, from a high of 5.0 to a low of 3.2. In Zimbabwe, after an initial decline from 4.0 to 3.4, PTSS QAC scores rose throughout Year 1 and remained consistently high during Year 2. PTSS QAC scores were only sporadically available during Year 3 owing to the reasons outlined above, but remained at acceptable levels (4.2 or above). Overall, combined QAC scores for the PTSS component were lower than VCT and CM during Year 1 of the intervention, and displayed greater fluctuations (Fig 2: Quality Assurance Scores for All Sites). Scores fell in the early months of the intervention to 3.4, and again towards the end of Year 1 to 3.4. Combined PTSS QAC scores were consistently high throughout Year 2. In Year 3, combined PTSS QAC scores fell to 3.7, but recovered to 4.7 by the end of the intervention. Across all sites, VCT and CM scores were higher than PTSS scores throughout Year 1. In Year 2, PTSS QAC scores had reached those of MVCT and CM, before falling below the other components again in the first half of Year 3. By the end of the intervention, PTSS QAC scores were again comparable to VCT and CM QAC scores.

## Discussion

Project Directors identified challenges experienced by study staff as (1) coping with the wide range of field activities and (2) the novelty of the CBVCT intervention. QAC score fluctuations were also associated with new staff hires or changes in staff responsibilities. The major effort put into developing and implementing the in-depth quality-assurance methodology has resulted in essential information on the successes and challenges of implementing this complex intervention. The overall 95% adherence to essential intervention delivery components indicates that the wide range of activities contained within the intervention can be successfully and faithfully implemented in resource limited settings. The QAC process has also been able to highlight challenges to implementation, especially around PTSS activities, and provided solutions to those challenges, primarily in the early identification of enhanced training needs. QAC data also serves an important purpose by monitoring consistency of component implementation. Of note, the application of feedback systems (e.g. site visits by the international intervention core team) were considered to be highly effective in remedying QAVC issues, though these could not be scientifically linked to changes in QAC results at the site level.

While the goal of QAC is to identify and correct non-adherence to the study protocol, it is also important to acknowledge elements that were performed particularly well and include these in the summaries as a way of motivating study staff. It is important that staff be given positive reinforcement in the areas where they are doing well and to be acknowledged for their valuable contribution to the project and to the team. These QAC procedures allow for the identification of skills training needs for VCT, PTSS, and CM staff, and relevant in-service skills trainings are discussed with the individual staff. In particular, VCT has been scaled up through-out African countries although very few report any attempts at QAC. Our experience in this large prevention trial is that such QAC is both (1) feasible and acceptable to both staff and participants and (2) associated with higher quality programmatic implementation.

The “diplomatic” QAC model presented under the Project Accept aegis is not without limitations. Given different social contexts, it may entirely appropriate to have greater flexibility across sites, as the present study did with permitting site team leaders and coordinators to set the frequency and structure of meetings to address QAC issues. However, this also introduces variability in how QAC issues are addressed, which could have some bearing on actual QAC across sites (as well as, for example, staff retention if some sites are better at this than others). Addressing some of these complexities and the need for additional research on how to assure QAC before implementing multi-site, multi-level interventions and assuring QAC once in the field remain key tasks for future related interventions. A further limitation to these results relates to the presentation of mean fidelity scores across the sites without associated data on competencies and specific adherence challenges or that seemed to explain fluctuations over time and differences across contexts. Such information, given the range of components and geographical areas covered by the current manuscript, was considered to be beyond the scope of this paper. Finally, there are numerous factors in addition to QAC that influence fidelity and the ultimate success of interventions and implementation strategies: although QAC is likely a useful tool for supporting and promoting high fidelity when HIV prevention interventions are implemented, one cannot make definitive causal statements in this regard.

## Conclusions

The implementation of a large-sale, evidence based HIV intervention requires extensive QAC to ensure implementation effectiveness, building on a ‘feedback loop’ whereby improvements in service delivery become both an iterative and an intuitive process. During Project Accept, ongoing appraisal of study staff by intervention component across sites helped to ensure consistent and high quality delivery of all intervention components, in keeping with the goals of the study protocol. In addition, for the first time in any kind of global health intervention, attempts were made to assess standards of service delivery from a diplomatic perspective. The development of the field of global health diplomacy has helped to drive recognition of the importance of culturally appropriate and culturally sensitive global health interventions, which both international and domestic Project Accept QAC staff monitored on a routine basis throughout the intervention. The primary findings of Project Accept also suggest that a community-wide multicomponent intervention of mobilization, HIV testing and support services can be both safe and feasible[17] and can significantly increase testing, particularly in men. The study also shows that the routine monitoring and assessment of services through QAC is an essential component of public health practice. In this context, QAC ensures staff fidelity to study procedures and is critical to the delivery of multi-site HIV prevention interventions. Both the findings and the associated methods of QAC for the Project Accept intervention could, therefore, usefully inform other interventions being “moved to scale”.

## Supporting Information

S1 Fig Zimbabwe Average Annual QAC Scores. (XLS)

S2 Fig Tanzania Average Annual QAC Scores. (XLS)

S3 Fig Vulindlela Average Annual QAC Scores. (XLS)

S4 Fig Soweto Average Annual QAC Scores. (XLS)

S5 Fig Thailand Average Annual QAC Scores. (XLS)
